# Cognitive Reserve Factors in a Developing Country: Education and Occupational Attainment Lower the Risk of Dementia in a Sample of Lebanese Older Adults

**DOI:** 10.3389/fnagi.2018.00277

**Published:** 2018-09-18

**Authors:** Hala Darwish, Natali Farran, Sarah Assaad, Monique Chaaya

**Affiliations:** ^1^Faculty of Medicine, Hariri School of Nursing, American University of Beirut, Beirut, Lebanon; ^2^Department of Public Health and Primary Care, Cambridge Institute of Public Health, University of Cambridge, Cambridge, United Kingdom; ^3^Department of Epidemiology and Population Health, Faculty of Health Sciences, American University of Beirut, Beirut, Lebanon

**Keywords:** dementia, cognitive reserve, neurodegenerative diseases, cognitive decline, cognitive impairment, lebanon

## Abstract

**Background:** Dementia secondary to neurodegenerative diseases is prevalent among older adults and leads to social, psychological and economic burden on patients, caregivers and the community as a whole. Cognitive reserve factors such as education, and mental stimulation among others were hypothesized to contribute to the resilience against age-related cognitive impairment. Educational attainment, occupation complexity, physical activity, and leisure activity are explored in the context of protecting the older adults' cognitive function. We investigated the cognitive reserve effect on dementia, cognitive decline and impairment, and global cognitive function.

**Methods:** This study is a secondary analysis of data from a cross-sectional, community-based cohort study that aimed at investigating factors associated with dementia and their prevalence. The sample was of 508 community based older adults in Lebanon, aged 65 years and above in addition to 502 informants designated by these older adults. Older adults and informants answered structured questionnaires administered by interviewers, as well as a physical assessment and a neurological examination. Older adults were diagnosed for dementia. Global cognitive function, depression, and cognitive decline were assessed.

**Results:** Older adults with dementia had lower levels of education, and attained lower occupational complexity. Factors such as high education, complex occupation attainment, and leisure activity, significantly predicted better global cognitive function. An older adult who attained high education levels or high complexity level occupation was 7.1 or 4.6 times more likely to have better global cognitive function than another who attained lower education or complexity level occupation respectively.

**Conclusion:** These results suggest that cognitive reserve factors ought to be taken into consideration clinically during the course of dementia diagnosis and when initiating community-based preventive strategies.

## Introduction

Dementia imposes considerable burden on populations throughout the world (Checkoway et al., 2011; Shah et al., 2016). Wimo et al. (2013) addressed the socioeconomic impact of dementia in 2010 and indicated that it has become the leading contributor to disability and need for care in aging adults. Dementia grew into a public health concern and efforts have been directed toward preventing, identifying, and reducing its risk (Larson et al., 2013; Prince et al., 2013; Shah et al., 2016). Dementia has a substantial impact on various aspects of the patients' life and can induce inability to carry out activities of daily living (Batista and Pereira, 2016). In Lebanon, the crude prevalence of dementia is 7.4% and the age-standardized prevalence of those above 65 years of age is 9.0%. Such figures make dementia prevalence in this country rank high within the global range of estimates but comparable to other Middle Eastern countries. For this age group the regional prevalence of dementia is as follow: 5–7 % in most world regions, 4.6 % in central Europe, 8.5 % in Latin America and 8.7 % in North Africa and the Middle East (Prince et al., 2013, 2015). Thus, it is projected that dementia will become a health crisis in Lebanon within the next 20 years (Phung et al., 2017).

Cognitive reserve has been explored in several population-based studies and found to be protective against brain damage and cognitive decline (Prince et al., 2012; Mondini et al., 2016). It is defined as the “differences in cognitive processes as a function of lifetime intellectual activities and other environmental factors. These alterations explain the differential susceptibility to functional impairment in the presence of pathology or another neurological insult” (Barulli and Stern, 2013). Consequently, the theoretical model suggests that individuals with high cognitive reserve levels will cope more efficiently with the same amount of brain damage as opposed to individuals with low levels (Cosentino and Stern, 2013). Factors such as reaching higher levels of education, engaging in complex occupations, and participating in more cognitively stimulating leisure activities enhance the cognitive reserve (Cosentino and Stern, 2013; Opdebeeck et al., 2016). These cognitive reserve-enhancing factors have been shown to be associated with reduced risk of dementia (Stern, 2009; Prince et al., 2012). They are attained at various stages across the lifespan, beginning with nourished environments and proper education during early life, to continuous mental, physical and social stimulation in middle and old age (Mondini et al., 2016; Wang et al., 2017).

The cultural and lifestyle diversity of middle-income countries posit interest concerning cognitive reserve (Wb, 2017). This is predominantly due to the variability in both the modular organization of the family structures and the sociocultural status of older adults across these countries (Katz et al., 2003; Prince et al., 2012). The components of cognitive reserve seem to differ across cultures. Prince et al. (2012) studied the effect of cognitive reserve on dementia incidence in several middle-income countries (Cuba, the Dominican Republic, Venezuela, rural and urban sites in Peru, Mexico, and China); their results support the protective hypothesis of cognitive reserve. However, unlike high-income countries, an association between occupational attainment and dementia incidence was absent in these countries (Prince et al., 2012). Exploring cognitive reserve factors in Lebanon is essential for developing culturally appropriate interventions. This study aimed to explore the association between several Cognitive Reserve (CR) indicators (education, occupation, physical activity, social activity, and leisure activity) and aspects of cognitive function status of a Lebanese sample aged 65 years and above. Dementia, cognitive decline, cognitive impairment, and global cognitive function represented the overall cognitive function status.

## Materials and methods

### Participants and procedures

This study is a secondary analysis of data collected for a cross-sectional study that aimed at investigating the prevalence of and factors associated with dementia among a **nationwide** community-based cohort of individuals older than 65 years randomly selected from all regions of Lebanon. Data collection for the larger study is still in progress - required sample size is 2500 persons (Phung et al., 2017). The sample size computed for this research was nested within the sample size of the national cohort. As such, the targeted sample size was 523 participants (335 from Beirut governorate, and 188 from Mount Lebanon governorate) (Phung et al., 2014, 2017). However, a minimum sample size of 500 persons was sufficient to allow a maximal error of ±2 with a 95% CI based on the available estimated dementia prevalence of 5% for North Africa and the Middle East at the start of the study (Phung et al., 2014, 2017). A total of 508 older adults were randomly selected from these two regions of Lebanon [Beirut (the capital city) and Mount Lebanon governorates] (Phung et al., 2017), in addition to 502 informants.

An older adult when selected was asked to identify a person who knew him/her best. This person could have been the spouse, a sibling, other relatives or an unpaid caregiver. All Participants gave informed consent before participating in the study and information was anonymous. The Institutional Review Board of the American University of Beirut approved this study, protocol number FHS.MC.19.

### Data collection measures

Older adults and their informants answered several structured questionnaires that were administered by trained interviewers (Broe et al., 1998). Moreover, older adults were subject to a physical assessment and a brief neurological examination (NEUROEX). Only the assessments relevant to the present analysis are detailed in this manuscript.

#### Cognitive status, mental health, physical, and neurological assessments

Dementia diagnosis, cognitive decline, cognitive impairment, and global cognitive function were included in the analysis. Presence or absence of depression was also examined, and physical health was assessed

Most participants in this study spoke only Arabic language (79.5%, *n* = 329), and a minority reported that they speak a language/languages other than Arabic (20.5%, *n* = 20.5). Therefore, dementia was diagnosed using the validated Arabic version of the one-stage 10/66 Dementia Research Group (DRG) which has 3 main components (Phung et al., 2014; Adi, 2017). The first component of this diagnostic assessment is the cognitive test battery which includes the Consortium to Establish a Registry of Alzheimer's Disease's (CERAD) animal naming tests with a modified 10-world list learning (Ganguli et al., 1996), in addition to the Community Screening Instrument of Dementia (CSI-D; more details can be found later; Hall et al., 1993). The latter has a validated predictive algorithm showing 83% specificity and 87% sensitivity (Phung et al., 2014), and combines educational and cultural fair which do not require reading or writing abilities (Hall et al., 1993). The second component of the dementia diagnostic assessment includes the informant interview of the CSI-D which indicates functional decline (Hall et al., 1993; Phung et al., 2014). The third and final component is the Geriatric Mental State (GMS) which applies a computerized algorithm (AGECAT) to identify organic brain syndrome (dementia), schizophrenia, neurotic and psychotic depression, and anxiety neuroses (Copeland et al., 1986). The 10/66 dementia diagnosis, is defined as scoring above a cut point of predicted probability for DSM-IV dementia syndrome from the logistic regression equation developed in the 10/66 international pilot study, using coefficients from the CSI-D, GMS/AGECAT diagnostic output, and modified CERAD 10-word list learning delayed recall score (Phung et al., 2014).

Cognitive decline and impairment were assessed using the 16-item Informant Questionnaire on Cognitive Decline in the Elderly (IQCODE). In this subjective measure, informants report changes in cognitive function in reference to the last 10 years A 5-point scale is used to rate function which ranges from 1 (much improved), to 5 (much worse). A score of 3 would imply no changes in cognition (Jorm, 2004; Phung et al., 2015). Cognitive decline is defined as a decline in cognitive skills from their premorbid level, and a score above 3 represents such changes in functioning, with higher scores suggesting more severe levels (Jorm, 2004). As such, and although different studies derived different cut-offs for impairment while using the terms interchangeably; (Jorm, 1994; Jorm et al., 1996; Harwood et al., 1997; Perroco et al., 2009) a cut-off of 3.34 (based on a Lebanese sample) was used here as indicative of cognitive impairment (i.e., more severe levels of cognitive decline; Phung et al., 2015).

The global cognitive function was derived from the “COGSCORE” of the CSI-D. This 32-item screening instrument includes an interview with the participant (cognitive assessment) and his/her informant (assessment of activities of daily living and functioning at work and in social relationships). Three summary scores are generated from the CSI-D: COGSCORE, an item-weighted total score from the participant cognitive test; informant score (RELSCORE), an unweighted total score from the informant interview; and discriminant function score (DFSCORE), a weighted score combining COGSCORE and RELSCORE. The global COGSCORE ranges from 0 to 34, and low scores have been associated with poorer cognitive function (Hall et al., 1993). Depression was assessed using the GMS (Copeland et al., 1986). Physical assessment and a brief neurological examination (NEUROEX) was also performed (Broe et al., 1998).

#### Proxy measures of cognitive reserve

Sociodemographic data were obtained using the 10/66 DRG questionnaires which were adapted to local contexts (ADI). This questionnaire **comprised** additional information used as proxy indicators of cognitive reserve. These included: level of education, the complexity of highest attained occupation, current employment status, social activity, leisure time activity and physical activity. Physical in addition to mental and social activities support cognitive activity and reserve (Cheng, 2016). These measures are described in the below sections, and details on how they were categorized are provided in Table 1.

**Table 1:** Proxy measures of cognitive reserve.

**Indicator**	**Description and categories**
Education	Four aggregate categories: none attained; low (5 to 9 years, pre-primary—intermediate); medium (12 years, secondary), and high (university or vocational training).
Occupation	1st categorization (2 levels): Currently employed or unemployed. 2nd categorization (5 levels): Complexity of highest ranked attained occupation: 0: never employed, 1: elementary occupations, 2: skilled and semi-skilled occupations, 3: services, technical, and associate professional occupations, and 4: managerial and professional occupations.
Current Physical Activity	1st categorization (4 levels): no activity beyond baseline activities of daily living (0 MET-min), low physical activity (1–499 MET-min), medium physical activity (500–999 MET-min) and high physical activity (≥1,000 MET-min). 2nd categorization (2 levels): active (low, medium, or high) or inactive (no activity).
Current Social Activity	Social integration: Frequency of social group meeting attendance were summed. No involvement was recorded as 0, occasional as 1, and regular as 2. The maximum score is 4, with higher scores indicating higher social integration levels (0–1 = most isolated; 2 = moderately isolated; 3 = moderately integrated; and 4 = most integrated). Social network size: represented by the average of number of children, relatives, friends, neighbors, and presence or absence of a spouse (presence = 1; absence = 0). Social network function: the rates of contact with family, friends, and neighbors (0 = never; 1 = less than 1 time per month; 2 = at least monthly; 3 = at least weekly; 4 = two to three times per week; 1 = daily) were summed. A composite score of social activity was derived by summing up the three indicators. Interquartile ranges were calculated to categorize the scores into three groups (low, medium, high).

##### Education

Attained educational levels were categorized according to the International Standard Classification of Education (ISCED-11). In addition to being internationally valid, the basic concepts and definitions of ISCED are comprehensive. It includes a full range of educational systems and classifies educational programmes by their content (UNESCO, 2012).

##### Occupation

Two aspects of occupation were taken into account for this study. The first is current employment status (employed vs. unemployed), and the second is the complexity of the highest ranked attained occupation (Correa Ribeiro et al., 2013). The latter was classified according to the required skill level to perform the work as indicated by the International Standard Classification of Occupations (ISCO-08). A skill level is defined as a function of complexity and range of tasks and duties to be performed in an occupation (ILO, 2012).

##### Physical activity

Participants' current physical activity levels were grouped based on the Metabolic Equivalent of Task -minute (MET-min) cut-offs used in the 2008 Physical Activity Guidelines for Americans (PAGAC, 2008; Hupin et al., 2015). Corrected MET values were calculated using the Harris-Benedict equation; the standard MET level (3.5 ml.kg^−1.^min^−1^) was adjusted for personal characteristics that may alter an individual's Resting Metabolic Rate (RMR) (i.e., age, sex, height, and body weight). This method was selected to adjust for possible underestimation of the true MET value, and misclassification of intensity categories (walking, moderate, and vigorous) in individuals that are overweight, older, low fit, or in women (Kozey et al., 2010). Physical activity was further classified into active or inactive. Detailed computation method may be found in Table 1 and Supplementary Material Data sheet 1.

##### Social activity

Current social integration and social network (size and function) constructs were quantified (Zunzunegui et al., 2003). Social integration represents the social involvement with other groups of individuals. It ranged from intimate to extended (religious, community, and social group meetings). The frequency of social involvement was derived. The average number of social ties was also computed to characterize the social network structure (Zunzunegui et al., 2003). Finally, social network function (frequency of social contact with friends, neighbors, etc.) was considered. A composite score of social activity was computed by summing up the three indicators.

##### Leisure activity

The frequency of current and previous (between the ages of 40 and 64 years) participation in gaming activities in addition to other hobbies (such as drawing, knitting, gardening, etc.) was derived. A composite score of current leisure activity, as well as previous frequency, were computed.

### Statistical analysis

Data from 508 older adults were included in this analysis (Prince and Jackson, 2009; Phung et al., 2017). Since the data had a non-parametric distribution (*p*-value for the Shapiro-Wilk test was less than 0.05), Mann Whitney and Kruskal Wallis statistical tests were performed. Spearman's rank order correlations were used to investigate the association between different cognitive reserve variables and dementia. Chi-square test was also used.

To explore the predictors of cognitive decline (two levels were used; no change in cognition and decline in cognition) and cognitive impairment; five binomial logistic regression analyses were performed for each outcome separately. Each regression included one set of cognitive reserve predictors (1: education, 2: complexity of highest attained occupation and current employment status, 3: current physical activity (total MET), 4: leisure activity (current and previous; continuous score), and 5: current social activity (network size, integration, and function). Significant predictors were entered in the final models while controlling for age and inserting depression as a potential confounder.

Ordered logistic regression analysis was performed for the global cognitive function as dependent variable while controlling for age and depression. Independent variables included the complexity of highest attained occupation (reference category: never worked), education (reference category: no education received), current physical activity (binomial), current and previous leisure activity (binomial), and current social activity (reference category: low social activity). The odds ratio for having better global cognitive function was calculated. All analyses were performed using the Statistical Package for Social Sciences, SPSS version 22 (IBM Corp., Armonk, NY).

## Results

### Descriptive data

Demographic data are presented in Table 2, results of the cognitive tests are in Table 3, and descriptive statistics of cognitive reserve constructs are in Table 4. The informants' mean age was 49.18 (±17.35), 68.1% females. The majority (41%) was a family member- son or daughter- of the older adults, 30.3% were the husband or wife and the rest 19.9% and 8% were close relatives or neighbor/friend respectively. Around 60% (58.2) of them co-resided with the older adults, 70% were married at the time of the study, 41.8% were employed, and 15.5, 16.5, and 25.3% completed primary, secondary, high school or more respectively.

**Table 2:** Demographic data.

	***Mean***	***SD***
Age	72.5	7.2
**GENDER**	***N***	***%***
Male	220	43.8
Female	282	56.2
**CURRENT EMPLOYMENT STATUS**		
Employed	150	30.5
Unemployed	341	69.5
**HIGH BLOOD PRESSURE (*****N*** = **491)**		
Yes	256	52.1
No	235	47.9
**HEART PROBLEMS (*****N*** = **491)**		
Yes	105	21.4
No	386	78.6
**STROKE (*****N*** = **491)**		
Yes	28	5.7
No	463	94.3
**DIABETES (*****N*** = **491)**		
Yes	157	32
No	334	68
**DEPRESSION**		
Yes	53	10.6
No	449	89.4
**DEMENTIA DIAGNOSIS**		
Yes	37	7.4
No	465	92.6
**COGNITIVE IMPAIRMENT (*****N*** = **501)**		
Yes	74	14.8
No	427	85.2
**COGNITIVE DECLINE (*****N*** = **493)**		
Yes	214	43.4
No	279	56.6

**Table 3:** Results of cognitive tests.

**Construct (and sub-domains)**		***Mean***	***SD***
**CSI-D**			
	COGSCORE (global cognitive function)	29.6	2.8
	RELSCORE	2.3	11.4
	DFSCORE	0.03	0.4
**CERAD**			
	Animal naming verbal fluency task	7	5.6
	10-word list learning task with delayed recall
	Learning trial 1 (score/10)	4.5	1.8
	Learning trial 2 (score/10)	5.2	1.7
	Learning trial 3 (score/10)	5.9	1.8
	Immediate recall (score/30)	15.55	4.6
	Delayed recall (score/10)	5	1.8
IQCODE (cognitive decline)		3.2	0.4

**Table 4:** Descriptive statistics of cognitive reserve constructs.

**Educational Attainment**	***N***	***%***
None	101	20.1
Low (5 to 9 years; pre-primary–intermediate)	279	55.6
Medium (12 years; secondary)	91	18.1
High (university levels and vocational training)	31	6.2
**HIGHEST RANKED ATTAINED OCCUPATION (*****N*** = **382)**		
Level 0 (never worked)	186	48.7
Level 1 (elementary)	94	24.6
Level 2 (skilled and semi-skilled)	58	15.2
Level 3 (services, technical, and associate professional)	22	5.8
Level 4 (managerial and professional)	22	5.8
**SOCIAL ACTIVITY**	***MEAN***	***SD***
Integration (*N* = 488)	1.5	1.3
Average Network Size (*N* = 491)	2	0.7
Network Function (*N* = 331)	12.6	1.9
Physical Activity (*N* = 413)	*Median*	*Range*
Total MET^*^	31.7	1666.7
	***N***	***%***
No activity	199	48.2
Low activity	185	44.8
Medium activity	19	4.6
High activity	10	2.4
**CURRENT GAMING ACTIVITY**	***N***	***%***
Never	322	65.6
Once per month or less	33	6.7
2–3 times per month	34	6.9
Once per week or less	32	6.5
2–3 times per week	34	6.9
Everyday	36	7.3
**PREVIOUS GAMING ACTIVITY**		
Never	274	55.9
Once per month or less	21	4.3
2–3 times per month	34	6.9
Once per week or less	52	10.6
2–3 times per week	58	11.8
Everyday	51	10.4
**CURRENT OTHER HOBBIES**		
Never	407	83.1
Once per month or less	17	3.5
2–3 times per month	10	2
Once per week or less	14	2.9
2-3 times per week	20	4.1
Everyday	22	4.5
**PREVIOUS OTHER HOBBIES**		
Never	357	72.7
Once per month or less	12	2.4
2–3 times per month	6	1.2
Once per week or less	23	4.7
2–3 times per week	45	9.2
Everyday	48	9.8
	***MEAN***	***SD***
Current Leisure Activity	1.6	2.3
Previous Leisure Activity	2.5	2.7

* *Estimated Metabolic Equivalent of Task (min/week)*.

### Cognitive reserve and different levels of cognitive performance

Participants diagnosed with dementia were less educated than those who were not. The complexity of highest attained occupation, and current physical activity were also different between the dementia groups, and negatively associated with dementia. The non-dementia group received more education, attained higher occupation and were more physically active. Current participation in gaming activity and other hobbies were higher in participants with no dementia.

Similar results were found when comparing participants who were cognitively impaired with those who were not. However, only social integration was negatively associated with cognitive impairment and higher among participants who were not cognitively impaired (Table 5).

**Table 5:** The cognitive reserve indicators in dementia vs. no dementia groups and cognitive impairment vs. no cognitive impairment group.

	**Dementia**					**Cognitive Impairment**				
	**U**	**Z**	**Sig**.	**Rho**	**Sig**.	**U**	**Z**	**Sig**.	**Rho**	**Sig**.
Education	5359	−4.233	0.000	−0.183	0.000	11836.5	−3.818	0.000	−0.169	0.000
Occupation^1^	3430.5	−2.646	0.008	−0.128	0.012	6760	−3.969	0.000	−0.178	0.000
Physical Activity^2^	2857	−3.386	0.001	−0.116	0.018	4831	−4.842	0.000	−0.148	0.003
Gaming Activity: Current	5173	−3.843	0.000	−0.152	0.001	10252.5	−5.133	0.000	−0.205	0.000
Gaming Activity: Previous	6521	−1.433	0.152	−0.058	0.203	11678	−3.198	0.001	−0.139	0.002
Other Hobbies: Current	6425.5	−2.173	0.03	−0.085	0.061	12394.5	−3.441	0.001	−0.145	0.001
Other Hobbies: Previous	7762	−0.011	0.991	0.011	0.809	14144	−1.079	0.281	−0.045	0.320
Social Network Size	6590.5	−1.483	0.138	0.083	0.067	13966.5	−1.009	0.313	0.043	0.343
Social Integration	6531	−1.339	0.181	−0.072	0.112	10073	−4.62	0.000	−0.212	0.000
Social Network Function	2457	−0.171	0.864	0.083	0.067	10669.5	−4.111	0.000	−0.043	0.343

1 *Complexity of highest ranked attained occupation*.

2 *Total MET*.

*Significance is two-tailed*.

*U, Mann-Whitney U; Z, z-score; Sig, significance level (p-value); Rho, Spearman's rank correlation coefficient*.

Kruskal-Wallis H and bivariate Spearman rho correlation tests were used to compare the different global cognitive function levels and each cognitive reserve variable (Table 6).

**Table 6:** Kruskal-Wallis test and bivariate Spearman's Rho correlation test.

	***N***	**Mean Rank**	**Chi-Square**	**df**	**Sig. [2-tailed]**	**Rho**	**Sig. [2-tailed]**
Education	Low	126	191.80	58.423	2	0.000	0.360^**^	0.000
	Medium	250	246.84					
	High	124	317.52					
	Total	500						
Highest Ranked Attained Occupation, Complexity	Low	106	129.88	66.132	2	0.000	0.409^**^	0.000
	Medium	200	200.43					
	High	75	252.24					
	Total	500						
Current Physical Activity: Total MET	Low	98	143.83	67.020	2	0.000	0.293^**^	0.000
	Medium	202	200.89					
	High	111	270.20					
	Total	411						
Gaming Activity: Current	Low	123	180.62	85.512	2	0.000	0.393^**^	0.000
	Medium	248	240.02					
	High	118	322.58					
	Total	489						
Gaming Activity: Previous	Low	122	195.26	50.430	2	0.000	0.323^**^	0.000
	Medium	248	237.2					
	High	118	310.62					
	Total	488						
Other Hobbies: Current	Low	122	215.11	28.518	2	0.000	0.234^**^	0.000
	Medium	248	242.76					
	High	118	278.54					
	Total	488						
Other Hobbies: Previous	Low	123	221.33	8.584	2	0.014	0.116^*^	0.01
	Medium	248	248.87					
	High	118	261.55					
	Total	489						
Leisure Activity: Current	Low	122	169.59	98.902	2	0.000	0.418^**^	0.000
	Medium	248	239.94					
	High	118	331.53					
	Total	488						
Leisure Activity: Previous	Low	188.02	169.59	45.923	2	0.000	0.307^**^	0.000
	Medium	242.82	239.94					
	High	306.42	331.53					
	Total	488						
Social Inte-gration	Low	122	237.00	4.994	2	0.082	0.102^*^	0.024
	Medium	247	235.55					
	High	117	267.05					
	Total	486						
Social Network Function	Low	50	266.32	1.32	2	0.507	0.052	0.348
	Medium	180	232.03					
	High	101	250.05					
	Total	331						

*Grouping variables: Global cognitive function (low, medium, high) and cognitive reserve indicators*.

** *Correlation is significant at the 0.01 level (2-tailed)*.

* *Correlation is significant at the 0.05 level (2-tailed)*.

*Z, z-score; Sig, significance level (p-value); Rho, Spearman's rank correlation coefficient*.

The results showed statistically significant differences in global cognitive function levels (low, medium, high) for each cognitive reserve variable. Education, the complexity of highest ranked attained occupation, current physical activity, current and previous gaming activity, in addition to current and previous other hobbies, all were associated with higher global cognitive function. The mean rank of all cognitive reserve categories increased as global cognitive function increased except for social integration and network function that did not reach statistical significance. Similarly, the variables education, complexity of highest ranked attained occupation, current physical activity, current and previous gaming activity, and current other hobbies were highest among the group with improved cognition, slightly lower in the group with no change in cognition, and lowest in the group with declined cognition. Social integration also varied across these groups **significantly and** was higher among the participants with better cognition.

### Predictors of cognitive function

Series of logistic regression analysis was performed to explore the association between the cognitive reserve indicators and cognitive status of the participants.

To start with, the effects of education, the complexity of occupation, current employment status, current physical activity, current leisure activity, current and previous gaming activities, hobbies, and current social integration and network function on the cognitive decline were analyzed. Age was controlled for in the analysis, and depression was entered as a potential confounder. This model was statistically significant, ${\text{X}}_{\left(10\right)}^{2}$ = 69.56, *p* < 0.001, and explained 31.7% of the variance (Nagelkerke R^2^) in cognitive status. Furthermore, 75% of the cases were correctly classified. All cognitive reserve factors were associated with a decreased likelihood of exhibiting cognitive decline. However, the Wald criterion demonstrated that only current leisure activity (*p* = 0.001) and current physical activity (*p* < 0.05) made a significant contribution to the prediction.

Then, the same analysis was performed to examine the association of the cognitive reserve indicators with cognitive impairment. This model explained 35.4% of the variance (Nagelkerke R^2^), was statistically significant (${\text{X}}_{\left(10\right)}^{2}$ = 78.919, *p* < 0.001), and correctly classified 89.5% of the cases. Current employment status and current leisure activity (*p* < 0.05) had a substantial impact.

Finally, while controlling for gender, age, and depression, an ordered logistic regression was performed to examine the effects of highest attained occupation complexity, education levels, current physical activity status, current leisure and social activities on global cognitive function. The model explained 46.4 % of the variance (Nagelkerke R^2^) and was statistically significant (${\text{X}}_{\left(13\right)}^{2}$ = 131.9, *p* < 0.000). We found that the odds of better global cognitive function increased with high education levels (*p* < 0.05), increased occupation complexity (*p* < 0.05) and leisure activity (*p* < 0.001). For instance, the older adult who once attained higher education levels or complexity level occupation was 7.1 and 4.6 [95% CI, 1.1 to 47.1; 1.2−17.9] times more likely to have better global cognitive function than another who attained a lower education level or comlex occupation respectively. There was no statistically significant association between low and medium education levels and social activity with global cognitive function. In Table 7 we report the odds ratio of having better global cognitive function while taking these variables into account.

**Table 7:** Ordered logistic regression exploring global cognitive function as an outcome and cognitive reserve indicators.

**Variables**	**Odds Ratio**	**95% CI**	**Wald X^2^**
High Education	7.1	[1.1–47.1]	4.1
High Occupation Complexity	4.6	[1.2–17.9]	4.8
Leisure Activity	3.7	[2.0–6.8]	17.6
Medium Occupation Complexity	2.5	[0.8–8.3]	2.3
Medium Education	2.2	[0.8–5.8]	2.6
High Social Activity	1.6	[0.7–3.5]	1.2
Physically Active	1.6	[0.9–2.9]	2.4
Low Occupation Complexity	1.0	[0.3–3.1]	0.0
Low Education	0.8	[0.4–1.7]	0.2

## Discussion

Our outcomes supplement the growing evidence on the cognitive reserve hypothesis. In line with previous findings conducted in high and low-income countries, the role of educational attainment, occupation complexity, and leisure activity is highlighted in the context of protecting the cognitive function of the elderly (Prince et al., 2012; Caffò et al., 2016; Litwin et al., 2016; Opdebeeck et al., 2016; Lavrencic et al., 2017; Wang et al., 2017). Distinct from the result of Prince et al. (2012), which was conducted in several middle-income countries (Prince et al., 2012), the profession type held by our participants was associated with the better cognitive function; higher complex occupations were attained more by subjects with no dementia and with better cognitive function in general. This result is in agreement with studies conducted in high-income countries (Opdebeeck et al., 2016).

Nonetheless, our findings should be interpreted with caution since most females in this sample never held a job. However, the women could have been significantly engaged in helping their kids with homework or leisure activities; such information could be used as a substitute for the traditional cognitive reserve measure of occupation. This information is worth collecting and measuring in future studies. Another limitation present in our study is possible measurement error of the cognitive reserve indicators. In this case, however, the measurement most likely led to underestimating the actual population effects.

Social activity constructs ought to be assessed using more comprehensive methods. Previous studies have implicated the protective role of social activity when referring to the cognitive function of the older adult (Zunzunegui et al., 2003). The reduced impact of this cognitive reserve indicator on cognition in our study was surprising since social activities have a significant influence on the mental and physical health among Lebanese individuals. Given the diverse and changing social structure in Lebanon, and since the older adults in this country are family oriented, investigating social typology and function as cognitive reserve factors may yield better insight to answering our question (Webster et al., 2014).

Further studies are needed to confirm and expand our results. Further studies are needed to confirm and expand our results. In addition to the cognitive reserve factors, we will consider collecting detailed family history, medical, and therapeutics exposure of the patients in future studies, as possible modifying factors. Environmental factors such as exposure to pollution, smoking or other toxins are also worth investigating in future studies.

Addressing cognitive reserve and function of the older adult in Lebanon may have favorable health outcomes. In the clinical setting, assessing cognitive reserve can aid in diagnosis and intervention (Cosentino and Stern, 2013). In the presence of brain damage, individuals with different cognitive reserve degrees will exhibit symptoms at various times Administering more robust assessment tools for patients with high levels of cognitive reserve may reduce the odds of misdiagnosis (Stern, 2012; Caffò et al., 2016). Similarly, the cognitive reserve may influence response to treatments of neurodegenerative diseases (Cosentino and Stern, 2013; Mondini et al., 2016). Recently, Mondini et al. (2016) examined the impact of different cognitive reserve levels on cognitive training administered to patients with dementia. Their results emphasized the predictive factor of cognitive reserve on the efficacy of neuropsychological rehabilitation training (Mondini et al., 2016). The cognitive reserve factor has so far translationally affected two notions. The first is upon the directions for developing and implementing comprehensive cognitive prescriptions in which nurses and other healthcare professionals contribute to. These prescriptions aim to enhance cognitive reserve and protect against cognitive decline (Vance et al., 2011). The second notion is policymaking adopted in some western European populations. Increasing cognitive reserve is suggested as a primary prevention method to decrease dementia occurrence. As compared to secondary and tertiary methods, this approach is implicated to have the most significant effect on reducing later dementia occurrence and disability. Balancing these three methods is recommended (Wu et al., 2016).

This study explored for the first time the relationship between proxy measures of cognitive reserve and various cognitive function indicators among the older adults in Lebanon. Older adults diagnosed with dementia had lower levels of education, occupational complexity, as well as current physical and leisure activity. The odds of the better global cognitive function at an older age were better as occupation complexity increased, higher education levels attained, and current physical and leisure activities performed.

The construct of cognitive reserve and the current findings associated with it offer a positive view toward addressing the increasing prevalence of dementia in Lebanon and how to protect cognitive function. Continuing education and adding complex tasks later in life, including after the age of 60 could be a possible intervention that might help aged people. This is worth investigating in future interventional research.

To our knowledge, our study is the first in this region to investigate the relationship between cognitive reserve and function in the older adult. More exploratory and interventional research in Lebanon and the Arab region is required to establish reliable conclusions about this concept in an attempt to increase the quality of the healthcare provided.

## Ethics statement

This study was carried out in accordance with the recommendations of the Federal Wide Assurance (FWA) For the Protection of Human Subjects, Institutional Review Board. The protocol was approved by the American University of Beirut's Institutional Review Board. All subjects gave written informed consent in accordance with the Declaration of Helsinki.

## Author contributions

HD contributed to conceptualization, design, data analysis and interpretation, drafting and revising of the manuscript for intellectual content. NF contributed to data analysis and drafting of the manuscript. SA contributed to data collection and revising the manuscript. MC contributed to data collection, data interpretation and revising of the manuscript for intellectual content.

### Conflict of interest statement

The authors declare that the research was conducted in the absence of any commercial or financial relationships that could be construed as a potential conflict of interest.

## Abbreviations

CR: Cognitive reserve
CERAD: Consortium to Establish a Registry of Alzheimer's Disease's
CSI-D: Community Screening Instrument of Dementia
DFSCORE: Discriminant function score
DRG: 10/66 Dementia Research Group
GMS: Geriatric Mental State
ISCED-11: International Standard Classification of Education
ISCO-08: International Standard Classification of Occupations
IQCODE: Informant Questionnaire on Cognitive Decline in the Elderly
RELSCORE: Informant score
MET-min: Metabolic Equivalent of Task -minute
NEUROEX: Neurological examination
RMR: Resting Metabolic Rate
U: Mann-Whitney U
Z: z-score
Sig: significance level (p-value)
Rho: Spearman's rank correlation coefficient
Df: degrees of freedom
Wald X^2^: Wald chi square test statistic
CI: Confidence Interval.
