# Supplementary material for: Cognitive Reserve Factors in a Developing Country: Education and Occupational Attainment Lower the Risk of Dementia in a Sample of Lebanese Older Adults
Source: Front Aging Neurosci. 2018 Sep 18;10:277. doi: 10.3389/fnagi.2018.00277 (PMC6153348; doi:10.3389/fnagi.2018.00277)
Supplement: Supplementary file 1 [file Data_Sheet_1.docx]

**APPENDICES**

**APPENDIX 1**

- MET values and Formula for computation of Met-minutes:
- Walking MET-minutes/week = 3.3 * walking minutes * walking days.
- Moderate MET-minutes/week = 5.0 * moderate-intensity activity minutes * moderate days.
- Vigorous MET-minutes/week = 8.0 * vigorous-intensity activity minutes * vigorous-intensity days.

→ a combined total physical activity MET-min/week can be computed as the sum of Walking + Moderate + Vigorous MET-min/week scores.

- Corrected MET value = MET value† * $\frac{3.5 {ml.kg}^{-1}\min^{-1}}{Harris-Benedict RMR {(ml.kg}^{-1}\min^{-1})}$
- Harris Benedict equation for RMR (kilocalories per day):

Male = 66.4730 + 5.0033 (Height cm) + 13.7516 (Weight kg) – 6.7550 (Age years).

Female = 655.0955 + 1.8496 (Height cm) + 9.5634 (Weight kg) – 5.6756 (Age years).

- To convert kilocalories per day obtained from the Harris Benedict equation2 to ml.kg^-1.^min^-1^, the following formula is used: kcal.day^-1^/1440 = kcal.min^-1^; kcal.min^-1^/5 = L.min^-1^; L.min^-1^/(weight kg)x1000 = ml.kg^-1.^min^-1^

†Compendium code: Ainsworth BE, Haskell WL, Herrmann SD, Meckes N, Bassett Jr DR, Tudor-Locke C, Greer JL, Vezina J, Whitt-Glover MC, Leon AS. 2011 Compendium of Physical Activities: a second update of codes and MET values. Medicine and science in sports and exercise. 2011 Aug; 43(8):1575-81.

**APPENDIX 2**

| **Significant different levels of cognitive reserve variables between groups.** | | | | |
| --- | --- | --- | --- | --- |
|  | **Dementia** | **Cognitive Impairment** | **Cognitive Decline** | **Global Cognitive Function** |
| **Education** | **•** | **•** | **•** | **•** |
| **Occupation Complexity** | **•** | **•** | **•** | **•** |
| **Physical Activity** | **•** | **•** | **•** | **•** |
| **Current Gaming Activity** | **•** | **•** | **•** | **•** |
| **Previous Gaming Activity** |  | **•** | **•** | **•** |
| **Current Other Hobbies** | **•** | **•** | **•** | **•** |
| **Previous Other Hobbies** |  |  |  | **•** |
| **Current Leisure Activity** | **•** | **•** | **•** | **•** |
| **Previous Leisure Activity** |  |  | **•** | **•** |
| **Social Network Size** |  |  |  |  |
| **Social Integration** |  | **•** | **•** |  |
| **Social Network Function** |  | **•** |  |  |

†Compendium code: Ainsworth BE, Haskell WL, Herrmann SD, Meckes N, Bassett Jr DR, Tudor-Locke C, Greer JL, Vezina J, Whitt-Glover MC, Leon AS. 2011 Compendium of Physical Activities: a second update of codes and MET values. Medicine and science in sports and exercise. 2011 Aug; 43(8):1575-81.
